# Telomere-to-telomere genome assembly of the wood tiger moth *Arctia plantaginis*

**DOI:** 10.1038/s41597-026-07316-x

**Published:** 2026-04-30

**Authors:** Eva L. Koch, Ian A. Warren, Melanie N. Brien, Johanna Mappes, Chris D. Jiggins

**Affiliations:** 1 Department of Zoology, University of Cambridge, Cambridge, UK; 2 School of Biodiversity, One Health & Veterinary Medicine, University of Glasgow, Glasgow, UK; 3 Organismal and Evolutionary Biology Research Program, Faculty of Biological and Environmental Sciences, University of Helsinki, Helsinki, Finland

## Abstract

The wood tiger moth, *Arctia plantaginis*, is an important model organism for the study of polymorphism in warning colour. One major colour locus has been identified, but studying its association with other traits as well as signatures of selection has been limited by the lack of a contiguous reference genome. A previous assembly consisted of more than 1000 scaffolds. Here, we present the first chromosome level genome assembly based on PacBio-HiFi data and linkage map information. The final assembly spans 589 Mb consisting of 31 telomere-to-telomere chromosomes with a N50 scaffold length of 21.2 MB. The BUSCO analysis (n = 5,760) indicated a high completeness of 98.1% with 97.5% present as single copy and 0.6% as duplicated. 17,456 genes were identified and 42.18% of the genome was classified as repetitive. This new assembly provides an important resource for future research using genomic tools for studying the genetic basis and evolutionary mechanisms underlying colour polymorphisms in this species.

## Background & Summary

**Fig. 1:**
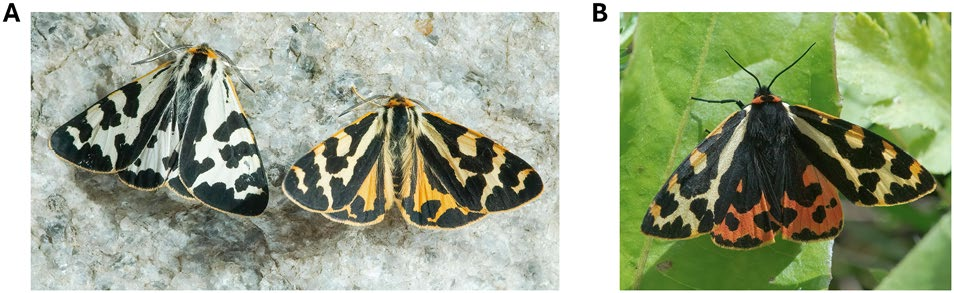
(**A**) White and yellow morphs of male wood tiger moths; **B:** female wood tiger moth. Images: Juhani Maamela.

The wood tiger moth, *Arctia plantaginis*, has become an important species for studying polymorphisms in warning colouration^1^. The species has a wide holoarctic distribution^2^ and is chemically defended, while using hindwing colour as a warning signal. Predators learn to associate the colour with unpalatability and subsequently avoid the same morphs. Despite the expected strong selective advantage of the most common form, stable colour polymorphisms persist in some populations. In Europe, males display two distinct morphs with either yellow or white hindwings, whereas females show continuous variation in hindwing colour ranging from yellow to red^3,4^ (Fig. 1).

The inheritance of male hindwing colour follows a Mendelian pattern^4,5^ with the white allele being dominant. The same colour alleles are present in females but do not generate polymorphism. The causal locus has been identified as a recent duplication of the *yellow-e* gene, resulting in an additional copy that is present only in white morphs and has been named *valkea*^6^.

There has been a long-standing interest in identifying potential associations between male hindwing colour and other traits, including chemical defences^7,8^, flight activity^9^, pheromones^10^, mating success^11–13^, and immune response^14^. These associations may help explain the maintenance of colour polymorphism: If male colour is part of a complex phenotype consisting of multiple traits, the two morphs may follow different strategies to optimise fitness, with neither morph being universally superior. Furthermore, the frequency of the yellow morph differs between populations: while relatively common in Finland, the yellow form is nearly absent in geographically close Estonian populations^2,15^ and also appears to suffer from reduced fitness in laboratory crosses. On the other hand, monomorphic yellow populations are found in other parts of Europe.

With advances in sequencing technology, it is now possible to study the maintenance of polymorphism at the genomic level by identifying the genetic basis of other traits under selection and their potential association with the colour locus. Furthermore, population genomic analyses can identify genomic regions under selection, reveal how selection varies across space and time, quantify gene flow between populations, and provide insights into demography, migration, and population expansion that may have impacted observed allele frequencies. A high-quality, continuous reference genome is key for these analyses and provides an important basis for future research, which is likely to advance our understanding of warning colour evolution not only in this system but also more broadly.

Two reference assemblies^16,17^ for this species, corresponding to the white and yellow haplotypes, were published in 2020^18^ using trio-binning of a heterozygous individual. These assemblies consist of more than 1,000 scaffolds, making it challenging to study the genomic context around the colour locus (or other regions of interest), which is located near the end of a scaffold.

Here, we present the first complete telomere-to-telomere assembly based on PacBio HiFi sequencing. The assembly has a BUSCO completeness score of 98.1% and very low duplication (0.6%). In total, we generated six de novo assemblies from individuals of an Estonian population and demonstrated how these assemblies can be used to detect structural variants that differ between populations with distinct warning colour frequencies. We also show how these assemblies allow a refined characterisation of the duplication breakpoints around the colour locus, the main genetic region of interest in this species.

The new assembly represents an important and valuable resource for future research on *A. plantaginis* and colour polymorphism in general. The complete chromosome-level assembly allows precise quantification of physical distances and linkage between this region and other loci identified in genome scans or association studies. Even without remapping older sequencing data, the reference enables ordering and localisation of previous scaffolds, thus improving interpretation of earlier results. It also provides higher resolution of the genomic region around colour locus.

## Methods

### Animal rearing

The samples were from a laboratory raised population (for details of stock maintenance see De Pasqual *et al*.^19^) of Estonian ancestry. In total, six samples, five males, one yellow and four white morphs, and one female, were sequenced (Table 1).

**Table 1:** Summary of sequencing statistics of the different samples.

Sample	Sex	Morph (male hindwing colour)	Total length [bp]	average read length [bp]	N50
CAM015214	male	white	15,058,490,434	19,988.40	19,900
CAM015215	female		18,007,740,664	18,267.70	18,184
CAM015216	male	yellow	15,422,188,436	18,518.60	18,418
CAM015217	male	white	18,647,518,592	16,052.10	16,075
CAM015218	male	white	20,711,323,074	15,997.10	15,985
CAM015219	male	white	15,455,884,501	19,445.20	19,393

### DNA extraction and sequencing

We extracted high molecular weight DNA using a modified version of the Qiagen MagAttract protocol 10.17504/protocols.io.6qpvr33novmk/v1. In addition, the DNA was eluted four times, with the last three elutions being performed at 37°C for 15 minutes. Each elution was tested for amount (Qubit fluorometer), purity (Nanodrop), and DNA integrity (Agilent Tapestation Genomic Screentape). For each individual, the best quality elutions were combined and cleaned using homemade magnetic beads to ensure that the amount, purity and concentration were sufficient for PacBio Sequencing (protocol: https://www.protocols.io/view/hmw-dna-extraction-using-magnetic-beads-b46bqzan). DNA was mixed with magnetic beads, incubated for 5 minutes, and put on a magnet until the liquid was cleared. The supernatant was then discarded and the beads washed twice with 80% ethanol. The beads were air dried and then redissolved in water and incubated for 30 minutes at 37°C with gentle shaking. The beads were placed on a magnet and, when clear, the supernatant was placed in a fresh tube. The DNA was again tested for amount, purity and DNA integrity as before. Library preparation and whole genome sequencing was performed by Novogene Bioinformatics Technology, China using one cell of a PacBio Revio system in HiFi mode. We obtained between 753,363 and 1,294,691 HiFi reads per sample with average read lengths between 16 and 20 kb (Table 1).

### Estimation of genome size

PacBio HiFi reads were used to estimate genome size and heterozygosity. K-mer frequencies were assessed using Jellyfish (v. 2.2.10)^20^ with a k-mer length of 21. The k-mer counts were exported to a histogram using jellyfish histo (-h 100000), setting the maximum count to 100,000 to include high-frequency k-mers. This histogram was then used as input for GenomeScope v1.0^21^ to estimate genome size. Genome size estimates inferred by GenomeScope based on k-mer spectra ranged from 566 to 578 Mb, close to the previously published genomes of 584.6 Mb for the white haplotype and 578.02 Mb for the yellow haplotype^18^. Heterozygosity estimates ranged from 1.83 to 2.24% (Figure S1; Table S1). The only yellow individual exhibited the lowest heterozygosity, as expected due to selective breeding in laboratory populations to increase the frequency of the rare yellow allele.

### Genome assembly

PacBio-HiFi long reads were de-novo assembled using Hifiasm (version 0.19.9-r616)^22^ with default parameters except from using -D 15 and -N 200 to improve the resolution of repetitive regions. This resulted in two phased genomes per individual. Assemblies from all samples showed a high quality with N50 ranging from 13 to 20 Mb and maximum scaffold lengths between 24 and 28 Mb (Table 2), which was higher compared to the published assemblies.

**Table 2:** Summary statistics of individual genome assemblies, the combined new assembly and the previously published assemblies for comparison (Yen *et al*. 2020).

sample	file	number of scaffolds	total length [bp]	minimum scaffold length [bp]	average scaffold length [bp]	maximum scaffold length [bp]	N50	L50	GC(%)	number of scaffolds with telomeres at both ends
**CAM015214**	CAM015214.1_ctg.fa	174	590,457,393	9,977	3,393,433.30	26,131,852	13,156,700	15	36.47	6
	CAM015214.2_ctg.fa	102	590,770,869	9,976	5,791,871.30	27,954,053	15,815,214	15	36.47	8
**CAM015215**	CAM015215.1_ctg.fa	251	617,375,702	19,980	2,459,664.20	24,522,243	17,048,143	15	36.82	15
	CAM015215.2_ctg.fa	88	567,978,423	23,340	6,454,300.30	25,119,091	19,036,279	13	36.62	15
**CAM015216**	CAM015216.1_ctg.fa	162	604,427,478	9,995	3,731,033.80	28,102,451	16,837,474	14	36.54	7
	CAM015216.2_ctg.fa	131	575,260,347	15,468	4,391,300.40	25,025,943	13,309,681	16	36.44	11
**CAM015217**	CAM015217.1_ctg.fa	805	645,124,644	10,211	801,397.10	25,320,923	16,961,450	16	37.63	15
	CAM015217.2_ctg.fa	106	580,137,160	18,683	5,472,992.10	27,942,518	19,441,888	13	36.46	15
**CAM015218**	CAM015218.1_ctg.fa	225	609,219,502	7,755	2,707,642.20	27,966,770	20,627,436	13	37.05	12
	CAM015218.2_ctg.fa	112	585,160,830	7,755	5,224,650.30	24,018,067	17,560,628	14	36.5	13
**CAM015219**	CAM015219.1_ctg.fa	283	628,409,855	27,020	2,220,529.50	28,274,369	15,142,282	16	36.77	7
	CAM015219.2_ctg.fa	143	577,774,801	9,988	4,040,383.20	25,126,691	13,036,657	17	36.57	8
**COMBINED NEW ASSEMBLY**										
	**New_Asm_Apla.fa**	32	588,994,248	15,767	18,406,070.30	28,274,369	21,249,390	13	36.47	31
**Previous References**										
	**White REFERENCE**	1,071	584,648,222	649	545,890	21,514,567	6,730,127	24	36.31	
	**Yellow REFERENCE**	1,052	578,016,695	1,343	549,445.50	24,414,994	9,770,308	18	36.58	

#### Identification of complete chromosomes and evaluating genome completeness

We aligned the new assemblies to the previously published assembly^18^ for the white morph (i.e., containing the duplicated *yellow-e* region responsible for white colour), hereafter called the W-reference, using minimap2 v. 2.28-r1209^23^ with parameters -cx asm5. The scaffolds of the W-reference had been assigned to 31 linkage groups (LGs)^6^, which is consistent with the number of chromosomes^18^. Within our new assemblies, several long scaffolds covered all W-reference scaffolds of a single LG, suggesting that they represent the entire chromosome or large portions of it. To assess whether these scaffolds correspond to complete chromosomes, we searched for telomere-specific motifs in all assemblies using SeqKit (v. 2.8.8). Telomere motifs were based on Lukhtanov and Pazhenkova^24^ including the main insect telomere motif (TTAGG) and telomere-specific retrotransposons of the SART and TRAS families. We examined the distribution of these motifs along the scaffolds to identify scaffolds with telomere sequences at both ends. Several scaffolds exhibited telomere motifs at both ends (Table 2), confirming that they represent complete chromosomes.

**Fig. 2:**
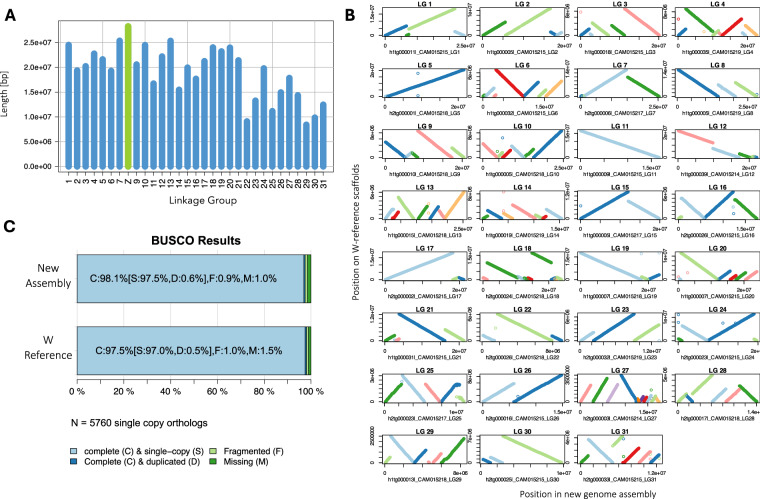
(**A**) Lengths of chromosomes, which correspond to the previously identified 31 linkage groups (LG) in the new assembly. We keep the naming by linkage groups to be consistent. Linkage group 8 corresponds to the Z chromosome (in green). (**B**) Alignment of scaffolds from the previous assembly of the white haplotype (Yen *et al*. 2020) to the new assembly by chromosomes (correspond to the previously identified linkage groups). The x-axes give the position on the scaffolds in our new assembly, the y-axis the position on the reference scaffold. Alignments of each LG are coloured by reference scaffolds. The new assembly shows a high synteny to the previous reference. It illustrates the order of the previous scaffolds along chromosomes and identifies a few errors in the previous alignment, for example an interspersed scaffold on LG18. (**C**) BUSCO completeness assessment of the new assembly and the previous white reference (“W reference”).

We combined scaffolds corresponding to complete chromosomes to generate a new reference genome (Table 3). We retained the original LG naming (Brien *et al*. 2023) to maintain consistency with previous publications, although the numbering does not reflect chromosome size order (Fig. 2A). Ordering the scaffolds of the previous W-reference along the newly identified chromosomes revealed overall very high synteny (Fig. 2B). The new assembly also allowed us to place W-reference scaffolds that were previously unassigned on the linkage map and to identify several inconsistencies: Some scaffolds were not contiguous but interspersed with other scaffolds (e.g., on LG 18 and on LG 28, Fig. 2B), while others combined sequences from two linkage groups. These results demonstrate that the new chromosome-level assembly can also help to accurately order and place scaffolds from previous references.

**Table 3:** Composition of the new assembly including origin of the individual scaffolds (naming: scaffold name, sample, linkage group).

LG	sample	scaffold	scaffold length [bp]	GC content (proportion)	repeats (proportion)	genes (proportion)	coding (proportion)
1	CAM015215	h1tg000011l_CAM015215_LG1	24,522,243	0.364	0.421	0.238	0.049
2	CAM015215	h1tg000005l_CAM015215_LG2	19,366,316	0.365	0.446	0.297	0.049
3	CAM015215	h1tg000018l_CAM015215_LG3	20,226,057	0.361	0.424	0.193	0.042
4	CAM015219	h1tg000035l_CAM015219_LG4	22,770,270	0.361	0.431	0.215	0.040
5	CAM015218	h2tg000001l_CAM015218_LG5	21,611,138	0.363	0.444	0.250	0.045
6	CAM015215	h1tg000032l_CAM015215_LG6	19,288,340	0.364	0.444	0.263	0.049
7	CAM015217	h2tg000006l_CAM015217_LG7	25,371,692	0.361	0.431	0.264	0.050
8	CAM015219	h1tg000005l_CAM015219_LG8	28,274,369	0.359	0.444	0.326	0.047
9	CAM015218	h1tg000010l_CAM015218_LG9	20,577,460	0.363	0.431	0.251	0.042
10	CAM015218	h1tg000005l_CAM015218_LG10	24,493,023	0.362	0.420	0.296	0.054
11	CAM015215	h1tg000009l_CAM015215_LG11	16,728,312	0.362	0.432	0.296	0.048
12	CAM015214	h1tg000039l_CAM015214_LG12	22,191,482	0.364	0.446	0.256	0.046
13	CAM015218	h1tg000015l_CAM015218_LG13	25,352,172	0.363	0.446	0.295	0.055
14	CAM015219	h1tg000019l_CAM015219_LG14	15,479,712	0.371	0.499	0.275	0.049
15	CAM015217	h1tg000005l_CAM015217_LG15	19,946,278	0.363	0.434	0.241	0.044
16	CAM015215	h2tg000026l_CAM015215_LG16	17,683,177	0.368	0.473	0.344	0.054
17	CAM015215	h2tg000002l_CAM015215_LG17	21,249,390	0.360	0.424	0.272	0.042
18	CAM015218	h2tg000024l_CAM015218_LG18	24,018,067	0.360	0.430	0.264	0.042
19	CAM015218	h1tg000007l_CAM015218_LG19	23,217,751	0.362	0.451	0.305	0.046
20	CAM015215	h1tg000007l_CAM015215_LG20	23,982,140	0.362	0.430	0.249	0.042
21	CAM015215	h1tg000031l_CAM015215_LG21	21,416,764	0.364	0.452	0.214	0.038
22	CAM015218	h2tg000026l_CAM015218_LG22	9,092,239	0.383	0.622	0.296	0.046
23	CAM015219	h2tg000032l_CAM015219_LG23	13,275,927	0.369	0.461	0.267	0.052
24	CAM015215	h2tg000023l_CAM015215_LG24	19,798,866	0.362	0.440	0.240	0.043
25	CAM015217	h2tg000023l_CAM015217_LG25	11,158,877	0.384	0.616	0.385	0.074
26	CAM015215	h2tg000016l_CAM015215_LG26	14,943,943	0.366	0.488	0.227	0.040
27	CAM015214	h2tg000003l_CAM015214_LG27	17,853,958	0.367	0.467	0.265	0.047
28	CAM015218	h2tg000017l_CAM015218_LG28	14,349,040	0.369	0.511	0.225	0.035
29	CAM015218	h1tg000013l_CAM015218_LG29	8,416,489	0.388	0.661	0.403	0.067
30	CAM015215	h2tg000025l_CAM015215_LG30	9,854,819	0.379	0.566	0.382	0.062
31	CAM015215	h2tg000033l_CAM015215_LG31	12,468,170	0.368	0.502	0.259	0.044
M	CAM015215	h1tg000185l_CAM015215_M	15,767	0.192	0.069		

Linkage groups follow the naming in Brien *et al*. 2023). M = mitochondrial genome.

Scaffolds corresponding to the mitochondrial genome were identified by aligning the assemblies to the published mitochondrial genome^25^. The final combined assembly consists of 31 telomere-to-telomere chromosomes and the mitochondrial genome, with a total length of 589 Mb and a GC content of 36.47%. Chromosome sizes ranged from 8.4 Mb (LG 29) to 28.1 Mb (LG 8, which represents the Z chromosome; Koch *et al*., in prep). N50 is 21.2 Mb and L50 (minimum number of scaffolds to cover 50% of the genome) is 13 (Table 3).

Genome completeness was assessed using BUSCO v.5.8.2 (Manni *et al*. 2021) based on the Lepidoptera_odb12 database (n = 5,760 single-copy orthologs). We also ran BUSCO on the previous W-reference genome using the same database for comparison (Fig. 2C). The analysis indicated high completeness of 98.1%, with a low level of duplication (97.5% single-copy, 0.6% duplicated; see Fig. 2C).

### Genome annotation and composition

We used Liftoff v1.6.3^26^ to lift over the previous annotation, ‘iArcPla.TrioW.annotation.gtf’^27^, from the W-reference^18^ to the new genome assembly, with the option to detect additional gene copies. A total of 17,456 genes of the 17,930 genes in the reference were successfully lifted over (97.4%) to the new assembly. The small proportion of genes that were not found in our assembly are distributed across all LGs in the W-reference and originated from 124 W-reference scaffolds, suggesting that no specific region is missing in our assembly. We are thus confident that our annotation is largely complete, although this approach could potentially miss Estonian population specific features. An annotation based on a lift-over from a reference from another population is probably less accurate than one incorporating Estonian transcriptomic data. However, the main aim of this study was the generation of a contiguous reference, and this annotation can provide initial guidance for evaluating functional impacts of any candidate loci identified in our new assembly, as well as insights into the overall genomic composition.

Overall, 27.0% of the genome is within genes and 4.7% within coding regions. The average length of single-copy genes was 8,946 bp, with an average of 6.5 coding sequences (CDS) per gene. Average CDS length was 238.5 bp.

The proportion of LGs within genes ranged from 19.3 to 40.3% and the proportion of coding regions between 3.5 and 7.3% (Table 3). There was no significant association between the coding proportion of a LG and its length (Spearman’s rank correlation = −0.19, P = 0.30) or between the proportion with genes and LG length (Spearman’s rank correlation = −0.34, P = 0.07), which was found to be a consistent pattern across Lepidoptera and specifically within the family of Noctuidea^28^.

**Fig. 3:**
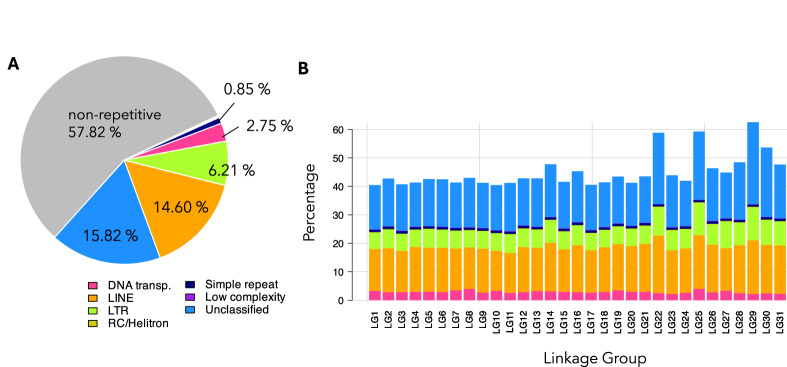
Proportion of different repeat classes genome wide (**A**) on each chromosome (**B**). DNA transp.: DNA transposons; LINE: Long Interspersed Nuclear Elements; LTR: Long Terminal Repeats; RC: Rolling-circle Eukaryotic Transposons.

We used RepeatModeler2 v.2.0.6^29^ to construct a de novo repeat library for the new assembly, which we then combined with ancestors and descendants of Insecta using the Python script famdb.py included in RepeatMasker v.4.1.8 (https://www.repeatmasker.org/) to access the Dfam v.3.9 database^30^. This combined repeat database was used to identify and classify repeats and to produce a genome with masked repetitive regions using RepeatMasker. Overall, 248.46 Mb (42.18%) of the genome was classified as repetitive. Among these regions, most were unclassified (n = 667,825; 15.82% of the genome). The largest group of classified repeats (n = 328,380; 14.60% of the genome) consisted of long interspersed nuclear elements (LINEs), followed by long terminal repeats (LTRs) and DNA transposons (6.21% and 2.75% of the genome, respectively; Table 4, Fig. 3A). Unclassified repeats and LINEs are generally common in Lepidoptera, whereas DNA transposons are relatively rare compared to other insect groups^31^.

**Table 4:** Summary of repeats elements identified in the genome.

Classification		Number	Length		Percentage in the genome	
Retroelements		510,540	122,534,781	bp	20.8	%
	SINEs:	0	0	bp	0	%
	Penelope:	0	0	bp	0	%
	LINEs:	328,380	85,976,213	bp	14.6	%
	CRE/SLACS	3,209	523,785	bp	0.09	%
	L2/CR1/Rex	81,602	19,949,804	bp	3.39	%
	R1/LOA/Jockey	123,127	42,102,578	bp	7.15	%
	R2/R4/NeSL	9,828	4,087,458	bp	0.69	%
	RTE/Bov-B	41,329	7,528,525	bp	1.28	%
	L1/CIN4	0	0	bp	0	%
	LTR elements	182,160	36,558,568	bp	6.21	%
	BEL/Pao	34,328	9,904,448	bp	1.68	%
	Ty1/Copia	3,457	1,579,100	bp	0.27	%
	Gypsy/DIRS1	28,928	8,654,932	bp	1.47	%
	Retroviral	0	0	bp	0	%
DNA transposons		69,273	16,182,672	bp	2.75	%
	hobo-Activator	6,243	1,242,919	bp	0.21	%
	Tc1-IS630-Pogo	33,913	10,650,543	bp	1.81	%
	En-Spm	0	0	bp	0	%
	MULE-MuDR	115	54,945	bp	0.01	%
	PiggyBac	5,408	481,418	bp	0.08	%
	Tourist/Harbinger	12,174	1,058,372	bp	0.18	%
	Other (Mirage, P-element, Transib)	373	184,593	bp	0.03	%
Rolling-circles		4,437	1,101,244	bp	0.19	%
Unclassified:		667,825	93,188,527	bp	15.82	%
Total interspersed repeats			231,905,980	bp	39.37	%
Small RNA		93,757	9,659,234	bp	1.64	%
Satellites:		0	0	bp	0	%
Simple repeats		118,428	4,985,859	bp	0.85	%
Low complexity		17,635	808,774	bp	0.14	%

SINE: Short Interspersed Nuclear Elements; LINE: Long Interspersed Nuclear Elements; LTR: Long Terminal Repeat.

**Fig. 4:**
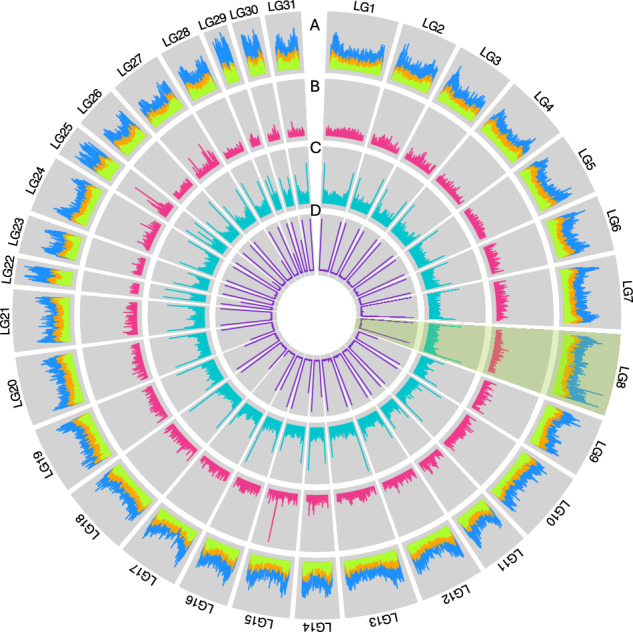
Genomic landscape of the 31 chromosomes. Tracks from outer to inner represent: (A) the distribution of the most common repeat classes (blue = unclassified; orange = long interspersed nuclear elements; green = long terminal repeats (see Fig. 3); (B) distribution of genes; (C) GC content; (D) distribution of telomere specific motifs. The Z chromosome (LG 8) is highlighted in green.

**Fig. 5:**
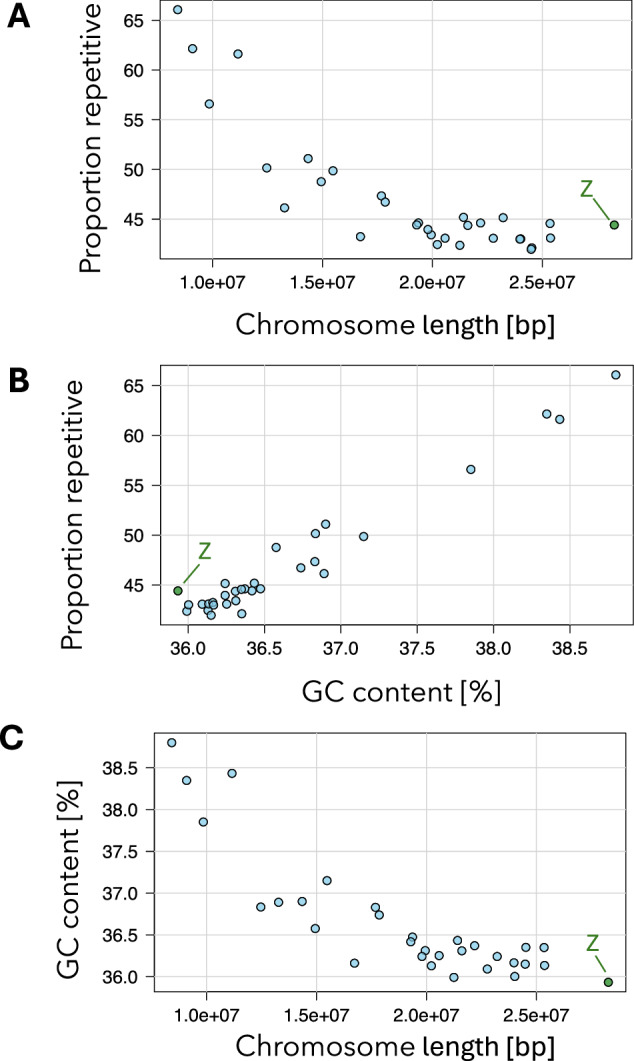
Relationship between repeat content and chromosome length (**A**); GC content and repeat density (**B**); and GC content and chromosome length (**C**). The Z chromosome is shown in green.

The average repeat content per LG was 47.0%, ranging from 42% to 66% (Fig. 3B). Overall, the distribution of repetitive elements across LGs was relatively even, although a few LGs (LG 22, LG 26, LG 29) showed higher proportions (>55%; Fig. 3B). On many LGs, the density of repetitive regions increased towards the chromosome ends, whereas the density of genes and coding regions decreased (Fig. 4), a pattern commonly observed in Lepidoptera^28^. We also found a negative correlation between repeat density and LG length (Spearman’s rank correlation = −0.80, P = 1.28e-06, Z chromosome excluded; Fig. 5A), consistent with patterns reported in other Lepidoptera^28^.

The genome wide GC content was 36.47%, which is similar to the published white (36.31%) and yellow (36.58%) references^18^. Variation between LGs was small with GC content ranging from 35.9 to 38.8% (Table 3). We found a strong positive association between GC content and the proportion of repetitive sequences per chromosome (Spearman’s rank correlation = −0.73, P = 7.51e-06; Z chromosome excluded, Fig. 5B) and a significant negative association (Spearman’s rank correlation = −0.78, P = 1.601e-06, Z -chromosome excluded) between GC content and LG length (Fig. 5C). GC content generally increased towards the ends of chromosome (Fig. 4).

### Male hindwing colour locus

**Fig. 6:**
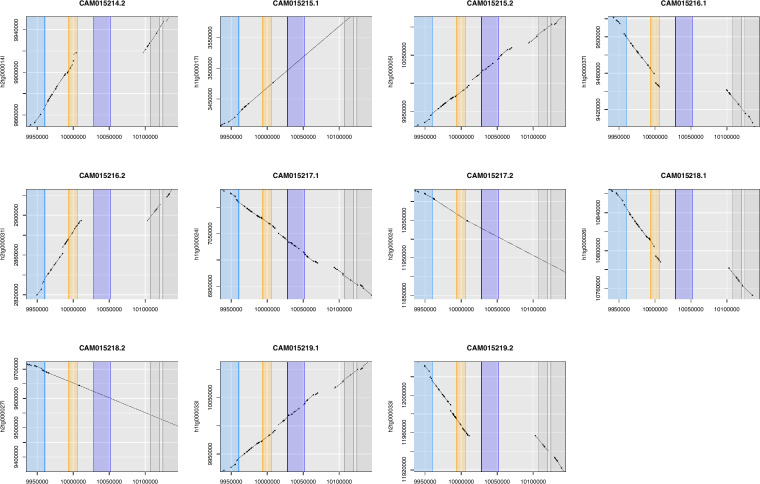
Alignment of all assemblies (two haploid assemblies per sample) to the new reference (we used CAM015214.1 for this scaffold) around the region with the male colour locus. Position in the new assembly is shown on the x-axis, position in the aligned assemblies on the y-axis. Dots indicate start and end of an alignment. Coloured areas refer to the position of genes: darkblue = *valkea* (gene jg1308 in the annotation), which is the gene responsible for white hindwing colour; orange = *yellow-g* (jg1309); lightblue = *yellow-e* (jg1310); grey areas: genes jg1306 and jg1307.

The locus determining male hindwing colour (the *valkea* gene) has been of particular interest in this species, but it has also been challenging to investigate. Since it resulted from a recent duplication event, a high proportion of reads from the ancestral *yellow-e* gene and its *valkea* copy cannot be mapped unambiguously, leading to many erroneously mapped reads when using short read sequencing. We aligned the haploid assemblies of all samples to both the W-reference and the new assembly, which contains the additional duplicated region including the *valkea* copy. This allowed us to genotype the samples and refine the breakpoints defining the duplicated region. Genotypes were inferred as two homozygous whites (CAM015215, CAM015217), one homozygous yellow (CAM015216), and three heterozygous individuals (CAM015214, CAM015218, CAM015219) (Fig. 6, S2). In the old W-reference (scaffold WW_tarseq_419_arrow), the duplicated region in the five haploid yellow assemblies started between 6,959,405–6,963,499 and ended between 7,073,701–7,075,118. In the new assembly (scaffold h1tg000039l_CAM015214_LG12), start breakpoints ranged from 10,004,082–10,011,215 and end breakpoints from 10,097,416–10,103,326. This slight variation might be caused by variations in repetitive sequences around the breakpoints, or inability of minimap2 to accurately align sequences in this region. Whereas one breakpoint in the old W-reference falls within the *yellow-g* gene (Figure S2), in the new assembly three of the five breakpoints are outside this gene, which is consistent with Brien *et al*.^6^ who found no expression differences of this gene between colour morphs.

### Structural variant analysis

**Fig. 7:**
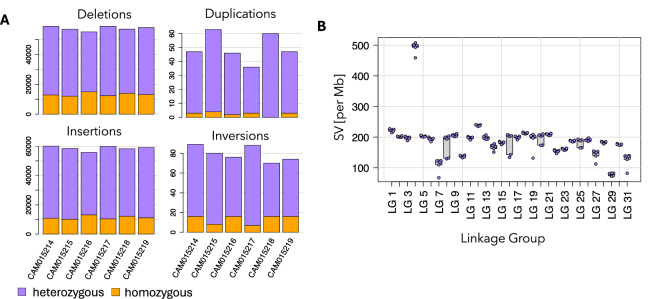
Number of different structural variants (SV) in the six samples relative to the white Finnish reference (**A**). Density of SVs (homozygous and heterozygous combined) across chromosomes in the five samples (**B**).

We used SVIM-asm (v1.0.3)^32^ to identify structural variants (SVs) in our new Estonian assemblies relative to the W-reference, which was based on a Finnish individual^18^. Having six samples allowed us to evaluate consistency in the type and distribution of structural variants. Insertions and deletions were the most frequently detected SVs in all samples when compared to the Finnish reference (Fig. 7A). The number of SVs was relatively consistent across samples (Fig. 7B, Table S2). We also observed an excess of SVs on scaffolds of LG4 in all samples (Fig. 7B). To evaluate the impact of different variant callers, we compared results of obtained from SV-asm, DeBreak v1.0.2^33^, and cuteSV v. 2.1.2^34^, following Liu *et al*.^35^. SVIM-asm uses an assembly-based approach, cuteSV is alignment-based, and DeBreak applies a hybrid strategy using read alignment combined with local assembly. The different tools mainly differed in the number of detected duplications, with DeBreak detecting many more than SVIM-asm and cuteSV (Table S3). The average sizes of insertions and deletions were similar across tools, but DeBreak detected larger duplications and inversions. The uneven distribution of SVs across LGs, with a high number on LG 4, was consistently detected by all variant callers (Figure S3).

## Data Record

Sequencing data generated for this study as well as the individual assemblies and the final composite assembly are available from the European Nucleotide Archive (ENA) under PRJEB106560^36^. The PacBio HiFi reads can be directly accessed on ENA under ERP187611^37^. The individual assemblies can found on ENA with the following accession numbers: GCA_978021575.1^38^, GCA_978021575.2^39^, GCA_978021285.2^40^, GCA_978021285.1^41^, GCA_978021605.2^42^, GCA_978021605.1^43^, GCA_978021585.2^44^, GCA_978021585.1^45^, GCA_978021655.2^46^, GCA_978021655.1^47^, GCA_978021665.1^48^, GCA_978021685.1^49^. The final assembly is available under ENA accession GCA_978021975.1^50^. The genome annotation is available from Zenodo^51^.

## Technical Validation

DNA purity was confirmed using a Nanodrop spectrophotometer and DNA integrity was assessed using the Agilent Tapestation. Average and N50 read length from PacBio HiFi sequencing was consistently 16 kB or higher indicating a high quality. Assembly completeness was evaluated using BUSCO and by aligning the assembly to the previous reference, which revealed a high synteny. Genome size estimates were consistent with predictions based on k-mer counting and with previously published references. The longest scaffolds corresponded to previously identified linkage groups and exhibited telomere-specific motifs at both ends, indicating that they represent full chromosomes. Genome composition, including repetitive element content, GC content, and their association with chromosome length, was consistent with general patterns observed in Lepidoptera. Structural variant distribution in the Estonian samples relative to the Finnish reference showed consistent patterns across six individuals and was confirmed using multiple variant-calling methods.

## Supplementary information

Supplementary Information

## Data Availability

PacBio raw HiFi reads and the assemblies are available from the European Nucleotide Archive (ENA) under project PRJEB106560 with the following accession numbers: PacBio-HiFi reads: ERP187611; individual assemblies: GCA_978021575.1, GCA_978021575.2, GCA_978021285.2, GCA_978021285.1, GCA_978021605.2, GCA_978021605.1, GCA_978021585.2, GCA_978021585.1, GCA_978021655.2, GCA_978021655.1, GCA_978021665.1, GCA_978021685.1. The final assembly is available under ENA accession GCA_978021975.1. Genome annotation is available from Zenodo (10.5281/zenodo.19385880).

## Code availability

No specific scripts or codes were used in this study. All software including versions and parameters used are described in the Methods.
