# Supplementary material for: Telomere-to-telomere genome assembly of the wood tiger moth Arctia plantaginis
Source: Sci Data. 2026 Apr 30;13:987. doi: 10.1038/s41597-026-07316-x (PMC13338207; doi:10.1038/s41597-026-07316-x)
Supplement: Supplementary file 1 — Supplementary Information [file 41597_2026_7316_MOESM1_ESM.pdf]

Supplementary Information

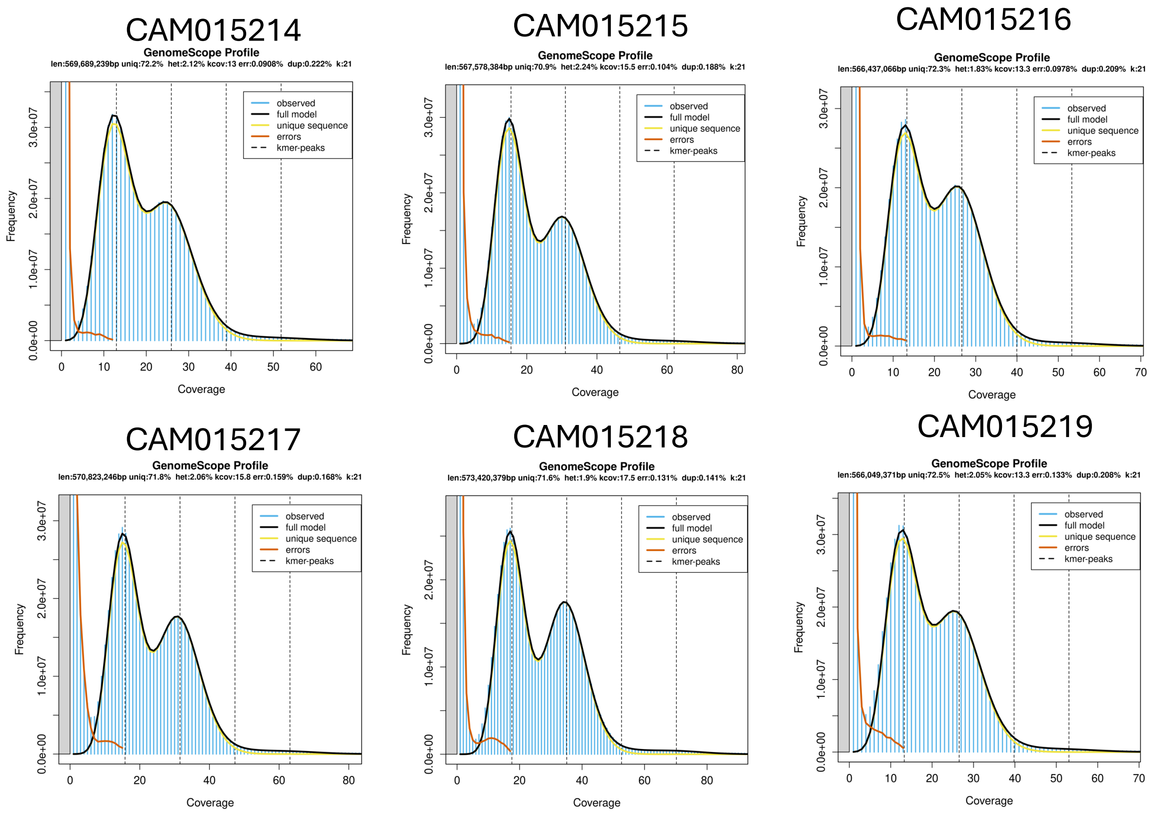

Figure S1 Genome size estimation by 21 K-mer frequency was estimated using GenomeScope. The vertical dotted lines indicate the coverage peaks for heterozygous, homozygous, and duplicated sequences separately.

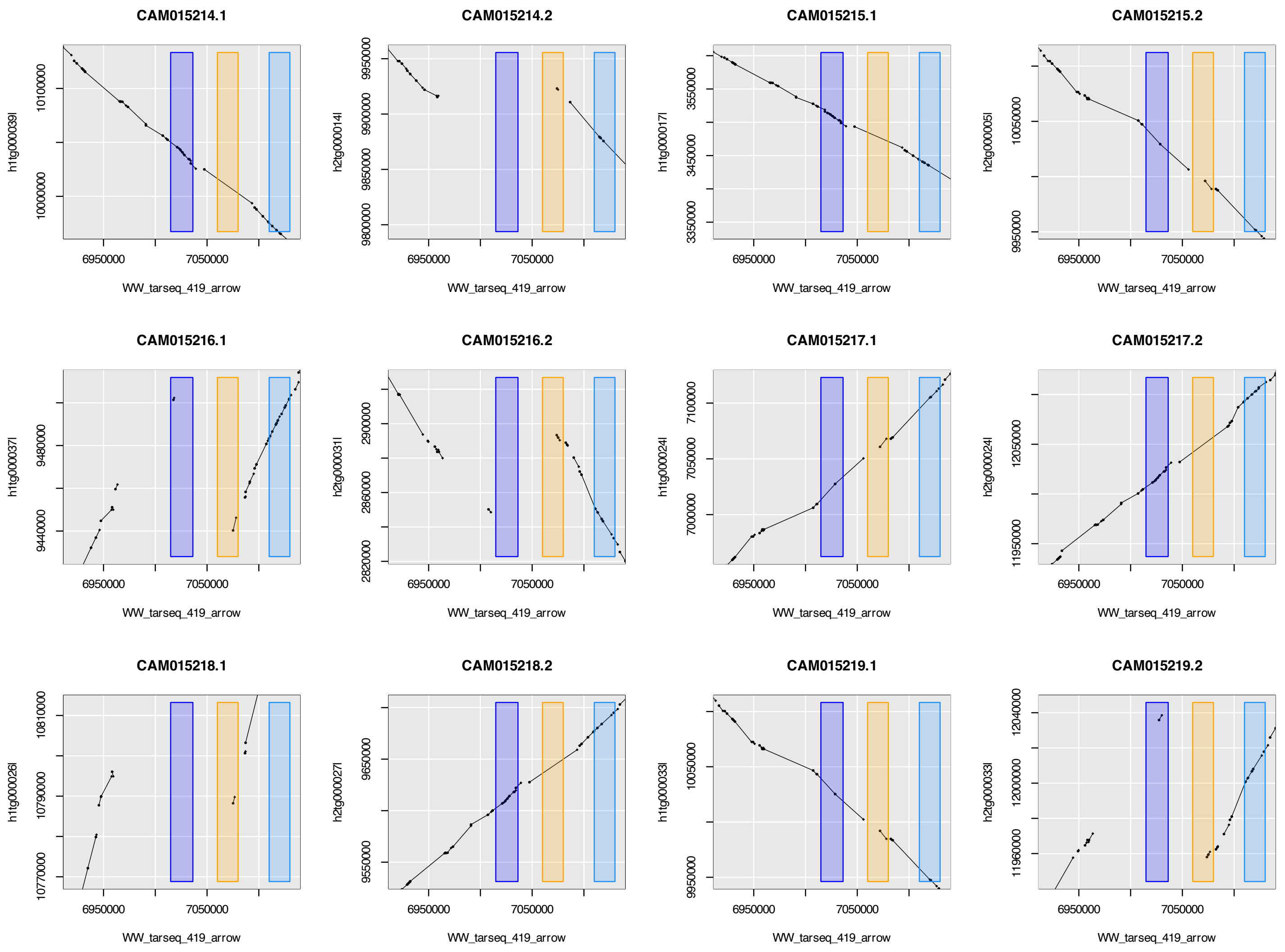

Figure S2 Alignment of new assemblies to the *valkea* region (on scaffold WW_tarseq_419_arrow) in the previous white reference (Yen et al 2020). Coloured areas refer to the position of genes: darkblue = *valkea* (gene jg1308 in the annotation), which is the gene responsible for white hindwing colour; orange = *yellow-g* (jg1309); lightblue = *yellow-e* (jg1310); grey areas: genes jg1306 and jg1307.

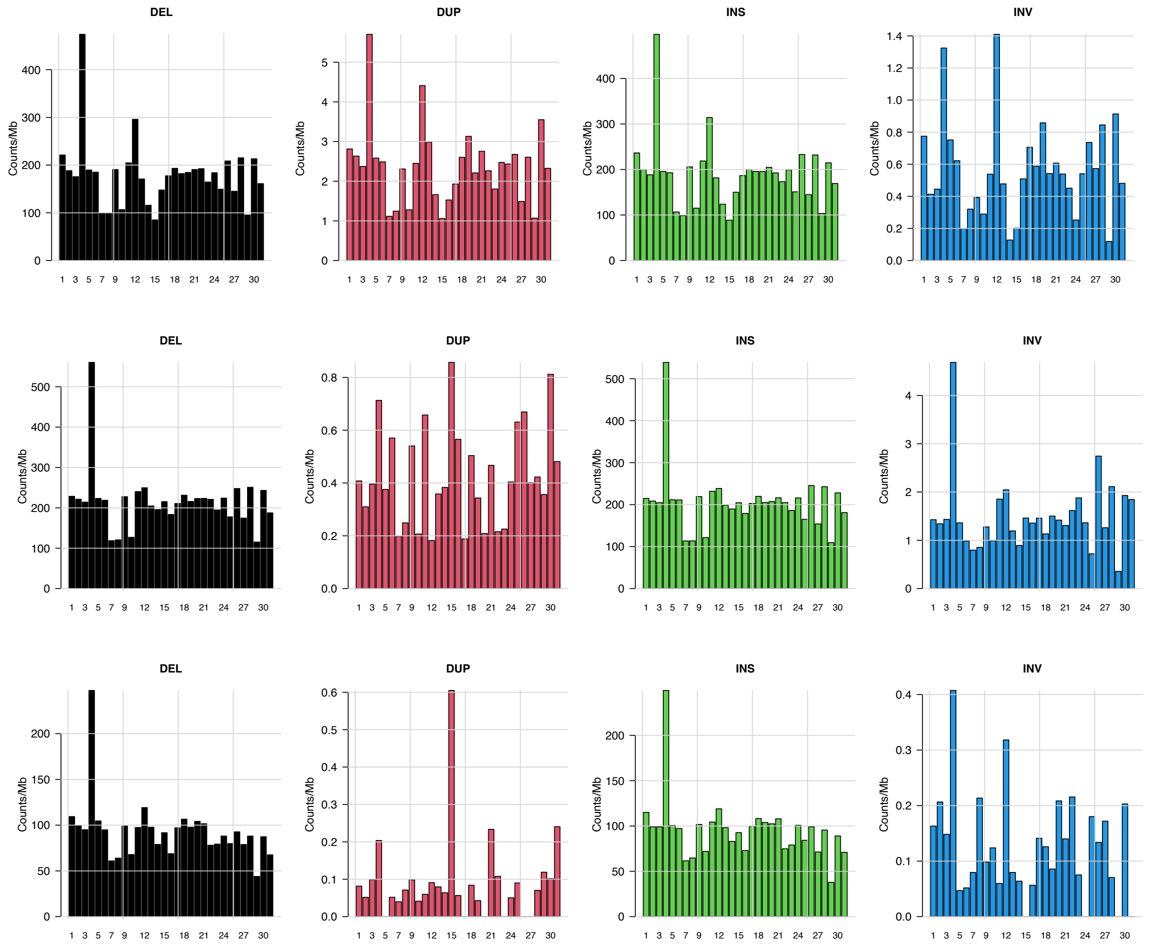

Figure S3 Frequency of detected structural variants (DEL: deletions; DUP: duplications; INS: insertion; INV: inversions) across linkage groups using three different variant callers. From top: DeBreak, cuteSV, SVIM-asm.

Table S1 Genome size estimation and characterization of the different samples based on GenomeScope.

	CAM015214		CAM015215		CAM015216		CAM015217		CAM015218		CAM015219	
**property**	**min**	**max**	**min**	**max**	**min**	**max**	**min**	**max**	**min**	**max**	**min**	**max**
**Heterozygosity**	2.12%	2.12%	2.24%	2.24%	1.83%	1.83%	2.06%	2.06%	1.90%	1.90%	2.05%	2.05%
**Genome Haploid Length [bp]**	569,585,685	569,689,239	567,495,224	567,578,384	566,348,346	566,437,066	570,734,766	570,823,246	573,307,895	573,420,379	565,949,271	566,049,371
**Genome Repeat Length [bp]**	158,359,238	158,388,029	165,010,704	165,034,884	156,608,982	156,633,515	161,028,206	161,053,170	163,081,549	163,113,546	155,779,154	155,806,706
**Genome Unique Length [bp]**	411,226,447	411,301,211	402,484,520	402,543,500	409,739,364	409,803,551	409,706,560	409,770,076	410,226,346	410,306,833	410,170,117	410,242,665
**Model Fit**	97.31%	99.27%	96.85%	98.95%	97.01%	99.01%	96.66%	98.66%	95.85%	97.84%	97.04%	99.01%
**Read Error Rate**	0.09%	0.09%	0.10%	0.10%	0.10%	0.10%	0.16%	0.16%	0.13%	0.13%	0.13%	0.13%

Table S2 Number and sizes of different detected structural variants using SVIM-asm. DEL = deletions; DUP:INT = interspersed duplications; DUP:TANDEM = tandem duplications; INS = inseretions; INV = inversions.

		Number					minimum					max					Size (mean)				
		DEL	DUP:INT	DUP:TANDEM	INS	INV	DEL *	DUP:INT	DUP:TANDEI	INS *	INV	DEL	DUP:INT	DUP:TANDEI	INS	INV	del.size	dup.int.size	dup.tandem.	ins	inv
**CAM015214**	homozygous	12902	0	3	10793	16	40	NA	610	40	390	18924	NA	6517	16753	4247	617.9	NA	2616.0	273.0	1320.4
	heterozygous	46079	2	42	49248	73	40	3528	56	40	255	44942	6010	98921	69821	57255	472.1	4769.0	4131.5	670.1	2670.4
**CAM015215**	homozygous	12060	0	4	10128	8	40	NA	924	40	390	19202	NA	6486	9476	3863	625.1	NA	4725.0	276.6	1721.8
	heterozygous	44895	2	57	48292	72	40	4793	88	40	195	55182	5990	40831	98731	3959	484.1	5391.5	4460.4	669.2	1140.4
**CAM015216**	homozygous	14966	0	2	13155	16	40		293	40	348	20605		494	27051	4906	608.5		393.5	369.2	1680.5
	heterozygous	40225	0	44	42708	60	40		79	40	227	55182		40149	75487	5690	467.6		3197.1	662.0	1166.3
**CAM015217**	homozygous	12441	0	3	10438	7	40	NA	297	40	265	19202	NA	5017	23952	1881	643.4	NA	3443.7	270.1	1232.7
	heterozygous	46508	2	31	49434	81	40	4567	78	40	213	38974	5990	16670	90675	7132	466.4	5278.5	1862.0	668.1	1395.6
**CAM015218**	homozygous	13992	0	0	12199	16	40		NA	40	303	18467		NA	18203	6622	609.2		NA	320.4	1353.9
	heterozygous	43037	0	60	46041	54	40		48	40	265	54092		39286	85172	6319	473.0		4278.1	658.8	1518.4
**CAM015219**	homozygous	13141	0	3	11183	16	40	NA	152	40	444	19202	NA	723	27051	3863	626.5	NA	403.7	294.9	1366.4
	heterozygous	44932	1	43	47934	58	40	4793	88	40	207	25318	4793	40831	98731	4920	467.9	4793	3569.8	664.1	1266.0

* 40 was default minimum value

Table S3 Comparison of different structural variant callers. Given are the mean, median numbers per structural variant as well as their minimum and maximum size.

	number				size mean				size median				size minimum				size maximum			
**Tool**	Deletions	Duplications	Insertions	Inversion	Deletions	Duplicat	Insertions	Inversion	Deletions	Duplicati	Insertion	Inversion	Deletions	Duplications	Insertions	Inversion	Deletions	Duplications	Insertions	Inversion
cuteSV	130068	280	124087	887	585.1708	5854.1	621.4565	1826.2661	170	2802.5	192	1105	30	33	30	212	84785	68671	18896	71272
DeBreak	108942	1444	115134	346	777.4016	62708	707.2994	268063.532	239	772.5	251	1518.5	48	48	48	284	8209926	11014091	13681	9779667
SVIM-asm	56955	58420	2/61	80	513.9329	4506.7	601.1393	1198.575	156	721	185	892.5	40	88	40	195	55182	40831	98731	3959
